# Supplementary material for: Metabolic and Obesity Phenotype Trajectories in Taiwanese Medical Personnel
Source: Int J Environ Res Public Health. 2022 Jul 4;19(13):8184. doi: 10.3390/ijerph19138184 (PMC9266400; doi:10.3390/ijerph19138184)
Supplement: Supplementary file 1 [file ijerph-19-08184-s001.zip › ijerph-1690613-supplementary.pdf]

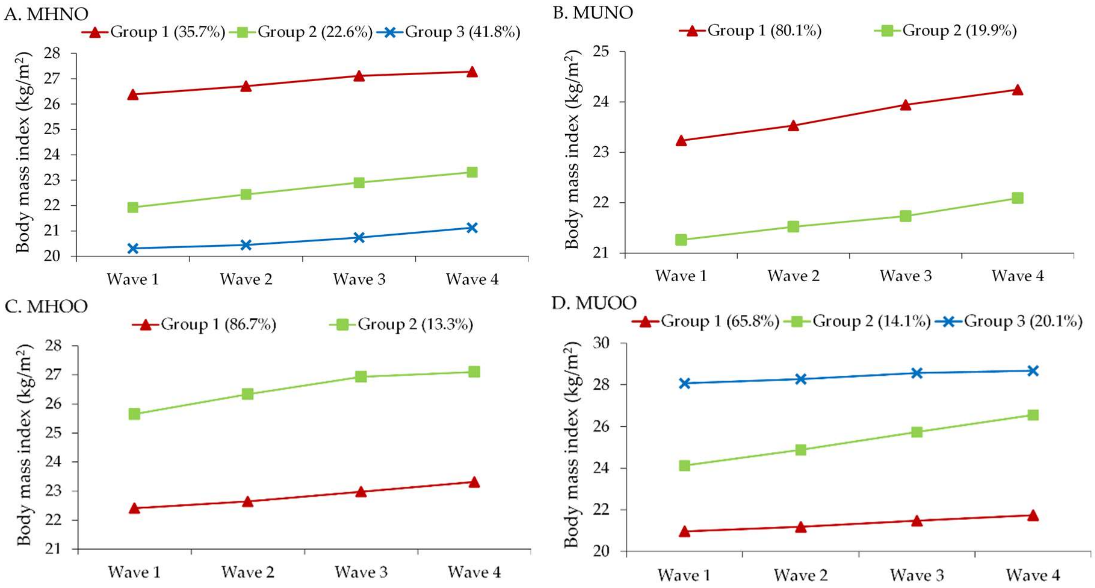

Supplementary Figure S1. The BMI changes over time in (A) MHNO, (B) MUNO, (C) MHOO, and (D) MUOO. MHNO, metabolically healthy non-overweight; MUNO, metabolically unhealthy non-overweight; MHOO, metabolically healthy overweight/obesity; MUOO, metabolically unhealthy overweight/obesity.
